# Plant defence responses in oilseed rape *MINELESS* plants after attack by the cabbage moth *Mamestra brassicae*

**DOI:** 10.1093/jxb/eru490

**Published:** 2015-01-06

**Authors:** Ishita Ahuja, Nicole Marie van Dam, Per Winge, Marianne Trælnes, Aysel Heydarova, Jens Rohloff, Mette Langaas, Atle Magnar Bones

**Affiliations:** ^1^Department of Biology, Norwegian University of Science and Technology (NTNU), NO-7491 Trondheim, Norway; ^2^German Centre for Integrative Biodiversity Research (iDiv) Halle-Jena-Leipzig, Deutscher Platz 5e, D-04103 Leipzig, Germany; Institute of Ecology, Friedrich Schiller University Jena, Dornburger-Str. 159, 07743 Jena, Germany; Molecular Interaction Ecology, Institute of Water and Wetland Research (IWWR), Radboud University Nijmegen, PO Box 9010, 6500 GL Nijmegen, The Netherlands; ^3^Department of Mathematical Sciences, Norwegian University of Science and Technology (NTNU), NO-7491 Trondheim, Norway

**Keywords:** *Brassica napus* (oilseed rape), defence cells, generalist, glucosinolate, jasmonates, myrosinase, plant–insect interaction, transcriptional profiling.

## Abstract

The Brassicaceae family is characterized by a unique defence mechanism known as the ‘glucosinolate–myrosinase’ system. When insect herbivores attack plant tissues, glucosinolates are hydrolysed by the enzyme myrosinase (EC 3.2.1.147) into a variety of degradation products, which can deter further herbivory. This process has been described as ‘the mustard oil bomb’. Additionally, insect damage induces the production of glucosinolates, myrosinase, and other defences. *Brassica napus* seeds have been genetically modified to remove myrosinase-containing myrosin cells. These plants are termed *MINELESS* because they lack myrosin cells, the so-called toxic mustard oil mines. Here, we examined the interaction between *B. napus* wild-type and *MINELESS* plants and the larvae of the cabbage moth *Mamestra brassicae*. No-choice feeding experiments showed that *M. brassicae* larvae gained less weight and showed stunted growth when feeding on *MINELESS* plants compared to feeding on wild-type plants. *M*. *brassicae* feeding didn’t affect myrosinase activity in *MINELESS* plants, but did reduce it in wild-type seedlings. *M*. *brassicae* feeding increased the levels of indol-3-yl-methyl, 1-methoxy-indol-3-yl-methyl, and total glucosinolates in both wild-type and *MINELESS* seedlings. *M*. *brassicae* feeding affected the levels of glucosinolate hydrolysis products in both wild-type and *MINELESS* plants. Transcriptional analysis showed that 494 and 159 genes were differentially regulated after *M*. *brassicae* feeding on wild-type and *MINELESS* seedlings, respectively. Taken together, the outcomes are very interesting in terms of analysing the role of myrosin cells and the glucosinolate–myrosinase defence system in response to a generalist cabbage moth, suggesting that similar studies with other generalist or specialist insect herbivores, including above- and below-ground herbivores, would be useful.

## Highlight

The larvae of *Mamestra brassicae* feed less and show reduced growth on plants with ablated myrosin cells, which raises questions about the role of defence cells in *Brassicaceae* plants.

## Introduction

The presence of the dual ‘glucosinolate–myrosinase’ system is a distinctive characteristic of the Brassicaceae family. Glucosinolates (β-thioglucoside-*N*-hydroxysulfates) are well known plant secondary metabolites that comprise a diverse group of sulfur-rich compounds and occur mainly in the order Brassicales (Bones and Rossiter, 1996; Kliebenstein *et al.*, 2005; Hopkins *et al.*, 2009; Sønderby *et al.*, 2010). Though the intact glucosinolates are shown to provide resistance to insect herbivores, their defensive potential is increased upon hydrolysis by the enzyme myrosinase (β-thioglucosidases; E.C. 3.2.1.147) (Kim and Jander, 2007; Ahuja *et al.*, 2010; Björkman *et al.*, 2011). Glucosinolate hydrolysis by myrosinases produces a range of compounds such as isothiocyanates, nitriles, epithionitriles, and oxazolidine-thiones, depending upon the glucosinolate structure, specifier proteins, and reaction conditions (Rask *et al.*, 2000; Rohloff and Bones, 2005; Bones and Rossiter, 2006; Wittstock and Burow, 2010). The protein cofactors that affect glucosinolate hydrolysis are epithiospecifiers (ESPs), nitrile-specifiers (NSPs), and thiocyanate-forming proteins (TFPs) (MacLeod and Rossiter, 1985; Lambrix *et al.*, 2001; Bones and Rossiter, 2006; Burow *et al.*, 2009; Kissen and Bones, 2009; Kong *et al.*, 2012).

Myrosinases are present in specialized ‘myrosin cells’ (Bones and Iversen, 1985; Bones *et al.*, 1991; Kissen *et al.*, 2009), which are dispersed throughout plant tissues. Immunocytochemical and *in situ* hybridization studies carried out on seeds of Brassicaceae species have shown that myrosinases are exclusively present in the myrosin cells of embryonic cotyledons and the radicle periphery (Bones *et al.*, 1991). The roles of myrosin cells and myrosinase have been well documented in several studies (Rask *et al.*, 2000; Kissen *et al.*, 2009; Textor and Gershenzon, 2009; Ahuja *et al.*, 2010).

The programmed cell death of myrosin cells was achieved during the seed development phase by directing the expression of an RNase (barnase) from a cell-specific myrosinase promoter (Borgen *et al.*, 2010). These plants have been named *MINELESS* because their myrosin cells (toxic mines) have been removed, leading to a dramatic reduction in myrosinase activity in seeds (Ahuja *et al.*, 2011). Additionally, the *MINELESS* plants have reduced amount of glucosinolate–myrosinase hydrolysis products, but higher amount of glucosinolates (Borgen *et al.*, 2010; Ahuja *et al.*, 2011).

The Lepidopteran insect *Mamestra brassicae* L. (Noctuidae) is a generalist that feeds on plants from at least 70 species and 22 families, of which members of the Brassicaceae and Chenopodiaceae are among the most preferred (McKinlay, 1992; Rojas *et al.*, 2000; Ulland *et al.*, 2008). It is common in Europe and Asia. Feeding by the caterpillars causes severe damage to the plants, and it is an economically devastating pest in agriculture (Ulland *et al.*, 2008). *M*. *brassicae* preference and performance are both affected by glucosinolate content and composition. *M*. *brassicae* larvae prefer to feed and perform best on gluconasturtiin-type rather than glucobarbarin-type *Barbarea vulgaris* plants (van Leur *et al.*, 2008). The larvae are also reported to benefit from a reduction in glucoraphanin, a predominant glucosinolate in *Arabidopsis* (Beekwilder *et al.*, 2008); and showed reduced performance on *B*. *oleracea* plants with high concentrations of the glucosinolate glucoiberin (Poelman *et al.*, 2009). Furthermore, silencing of the foliar myrosinase genes *TGG1*/*TGG2* enhanced growth of *M*. *brassicae* larvae after feeding on *Arabidopsis* plants (Zheng *et al.*, 2011).

Here, we took an exclusive opportunity to study the responses of *B*. *napus* wild-type cv. Westar and *MINELESS* plants after they had been attacked by the larvae of *M. brassicae*. The aim was to find out what happens in plants that lack defence cells, such as myrosin cells; or in other words what kind of defence responses are regulated in plants with a modified glucosinolate–myrosinase defence system upon attack by the generalist insect herbivore *M*. *brassicae*. Since glucosinolate levels were higher and glucosinolate–myrosinase hydrolysis products were lower in *MINELESS* than the wild-type, we also evaluated how *M*. *brassicae* larvae develop on wild-type and *MINELESS* plants. The results are presented as a comparative study of wild-type vs *MINELESS* plants in response to insect herbivory by larvae of *M*. *brassicae*.

## Materials and methods

### Plant and insect rearing

The *B. napus* wild-type cv. Westar and *MINELESS* seeds were germinated in soil (Borgen *et al.*, 2010; Ahuja *et al.*, 2011), and the plants were grown under greenhouse conditions (S3 security class), with a 16h photoperiod. The day and night temperatures were 21 and 18°C, respectively, at a light intensity of 70–80 μmol m^–2^ s^–1^. The eggs of *M. brassicae* were kept at 21°C/16°C, light 16h/dark 8h.

### Insect no-choice feeding experiments

Seven-to-nine-day-old wild-type and *MINELESS* seedlings were infested with neonate *M. brassicae* larvae (Cabbage moth; Laboratory of Entomology, Wageningen University) (Supplementary Table S1). Neonate larvae were weighed to assess their average starting weight and then distributed over pots (four seedlings per pot) of each of the wild-type and *MINELESS* seedlings. Each seedling was infested with one neonate larvae by placing it on one of the two cotyledons. The larvae were trapped in cages made of opaque plastic sheets. The seedlings were completely enclosed by placing one side of the cage on a pot and covering the other side with a muslin cloth. The experiments were repeated twice. In a first experiment, the insect no-choice feeding assay was performed for 12 days by taking insect weights at four time points (day 3, 8, 10, and 12) (Supplementary Table S1). In a second experiment, the same was performed for 12 days by taking insect weights at two time points (day 7 and 12) (Supplementary Table S1). Larvae were given new seedlings at each time point. The above-ground tissue (cotyledons + hypocotyl) from each seedling was scanned and comprised one biological observation. The area of scanned tissue was calculated with software Compu Eye, Leaf & Symptom Area (Bakr, 2005). To account for the damage to tissue during the experiment, the amount of consumed area was calculated by comparison with the control tissue.

### Induction experiment

The effects of larval attack on wild-type and *MINELESS* seedlings were measured in the following way: 10-day-old *M. brassicae* larvae were kept on 6–7-day-old seedlings of wild-type and *MINELESS*, respectively (Supplementary Table S1). In order to retain the larvae, the pots (four seedlings per pot) were enclosed in cages. Controls were treated in the same way, without insects. Pots with control (non-infested) and *M. brassicae*-infested seedlings were kept under the same conditions. The control and *M. brassicae*-challenged seedlings were harvested after 24h of infestation. Cages and larvae were removed and the above-ground tissue from seedlings was harvested and flash frozen in liquid nitrogen. These samples were homogenized to a fine powder and used for myrosinase activity assays, glucosinolate analysis, microarrays, and qRT-PCR experiments. Fresh tissue was used for extraction of glucosinolate–myrosinase hydrolysis products.

### Myrosinase activity and protein assays

The extraction of myrosinase and specific myrosinase activity were measured from control and insect-challenged samples following previous methodology (Borgen *et al.*, 2010; Ahuja *et al.*, 2011). The wild-type and *MINELESS* seedlings were crushed and the proteins were extracted in 100 µl of imidazole-HCl buffer (10mM, pH 6.0). The myrosinase activity was measured using the GOD-Perid assay, as described previously (Bones and Slupphaug, 1989; Borgen *et al.*, 2010). In order to calculate the specific myrosinase activity, the total protein content of samples was measured using Bradford reagent (BioRad Laboratories, UK). The specific myrosinase activity is described as nmol glucose generated min^–1^ mg^–1^ protein.

### Glucosinolate analysis

Glucosinolate analysis was performed as described previously (van Dam *et al.*, 2004). Lyophilized, finely ground above-ground tissue was dissolved in 1ml 70% MeOH in water (v/v) in a 2ml Eppendorf tube, vortexed, and immediately boiled for 5min to inactivate any remaining myrosinase. The tubes were placed in an ultrasonic bath for 15min and centrifuged (10min, 10 000rpm). The extraction was repeated for the pellet, but with the boiling step omitted. Both supernatants were combined per sample and applied to a DEAE-Sephadex A 25 column, desulphated with arylsulphatase (Sigma, St. Louis, IL, USA), and separated on a reversed phase C-18 column on HPLC with a CH_3_CN–H_2_O gradient. Glucosinolate detection was performed with a Photo Diode Array (PDA) detector (200–350nm) with 229nm as the integration wavelength. Sinigrin (2-propenylglucosinolate) was used as an external standard. The response factors at 229nm from three sources (Buchner, 1987; EC, 1990; Brown *et al.*, 2003) were used to calculate the concentrations of the glucosinolates. Desulphoglucosinolate peaks were identified by comparing HPLC retention times and UV spectra with standards kindly provided by M. Reichelt (MPI Chemical Ecology, Jena, Germany) and a certified rape seed standard (Community Bureau of Reference, Brussels, code BCR-367R).

### Analysis of glucosinolate–myrosinase hydrolysis products

Glucosinolate hydrolysis products were analysed as described previously (Ahuja *et al.*, 2011). The seedlings were crushed with a glass rod in MQ H_2_O in a 2ml screw-top vial with a PTFE/silicone septum. The mixture was left for 10min at ambient temperature for hydrolysis. A mixture of 0.5ml hexane:dichloromethane (3:2) with an internal standard (12 μg butyl-isothiocyanate) was injected through the septum into the vial, and the sample was vortexed for 50 s. After centrifugation at 3 100rpm for 2min, the solvent phase was pipetted into a 2ml screw-top vial with a PTFE/silicone septum, and concentrated under nitrogen flow to a volume of 50 μl. Agilent 6890/5975 GC-MS (Agilent Technologies Inc., Palo Alto, CA) was used for all analyses. Mass spectra were acquired in EI mode, and a mass range of m/z 39–250 was recorded. The compounds were quantified as described previously (Ahuja *et al.*, 2011).

### RNA isolation, cDNA synthesis, microarray experiments, and data analysis

Microarray experiments were performed to compare the expression profiles of wild-type controls against *M. brassicae*-challenged wild-type seedlings, and the *MINELESS* controls against *M. brassicae*-challenged *MINELESS* seedlings by taking four biological replicas of each. All procedures were followed as described previously (Kusnierczyk *et al.*, 2008). Statistical analysis was done using R (version 2.10.1), and the Linear Models for Microarray Data (LIMMA) package (Smyth, 2004). The data sets were log-transformed and normalized using the print tip-loess approach. Within-array replicated measurements for the same gene were merged by taking the average between replicates. No background subtraction was performed. Probe-specific dye effects were identified and genes with a strong dye bias were removed. Adjusted *P*-values were calculated using the Benjamini-Hochberg step-up procedure, controlling the false discovery rate. Genes with an adjusted *P*-value <0.05 were considered to be significantly differentially expressed. To evaluate the difference in responses between *M. brassicae*-challenged wild-type seedlings and *M. brassicae*-challenged *MINELESS* seedlings, a LIMMA contrast analysis was performed using control (non-infested) plants as a common reference. The genes were grouped depending on the biological process with which they are mainly associated using the Arabidopsis Information Resource (TAIR) database (http://arabidopsis.org/index.jsp) (Poole, 2008), GenomeNet (http://www.genome.jp), and cited literature. The microarray data were deposited in the Gene Expression Omnibus (http://www.ncbi.nlm.nih.gov/geo/) with accession number GSE40932, and are compliant with the MIAME guidelines.

### Quantitative PCR

Quantitative PCR was performed as described previously (Kusnierczyk *et al.*, 2011), with minor modifications. The cDNA was synthesized from 1 µg total RNA using the QuantiTect® Reverse Transcription Kit (Qiagen). The qRT-PCR was carried out using the LightCycler 480 SYBR Green I Master mix (Roche Applied Science, Mannheim, Germany), following the manufacturer’s instructions. PCR was performed in a LightCycler 480 as follows: (i) Pre-incubation for 5min at 95°C; (ii) 45 amplification cycles consisting of 95°C for 10s, 55°C for 15s, and 72°C for 10s; (iii) 95°C for 5 s, 65°C for 1min, followed by a melting curve analysis by heating from 65°C to 97°C with a ramp rate of 2.2°C s^–1^. Each 20 μl reaction contained 0.5 μM of each of forward and reverse primer and a cDNA quantity corresponding to 0.05 μg of total RNA. Cp values and melting curves were calculated by the LightCycler 480 analysis programs using the 2nd derivative maximum function and exported in text format. PCR efficiencies were determined employing LinReg PCR (Ramakers *et al.*, 2003). Relative expression ratios were calculated using the Relative Expression Software Tool (REST 2008) (Pfaffl *et al.*, 2002). All biological replicas were tested with each primer set. The primer sequences for *Brassica* genes representing *Arabidopsis* homologues (which were regulated in the microarrays) are described and pooled (Supplementary Tables S2 and S3).

### Statistical analysis of feeding experiments

Statistical analyses were performed using R language (R Development Core Team, 2011), and IBM SPSS Statistics version 20. Nonparametric tests were used, since data in general could not be assumed to be normally distributed (based on Anderson-Darling normality tests and quantile-quantile plots). For the no-choice experiments the differences between the insect weight gain of larvae retrieved from the wild-type and *MINELESS* plants on days 3, 7, 8, 10, and 12 were analysed using Wilcoxon Mann-Whitney tests, where *P* < 0.05 was considered significant. For the induction experiments, the levels of myrosinase activity, glucosinolates, and glucosinolate hydrolysis products among four different groups (wild-type control, wild-type *M*. *brassicae*, *MINELESS* control, and *MINELESS M*. *brassicae*) were analysed by a non-parametric version of one-way analysis of variance, the Kruskal-Wallis test (where *P* < 0.05 was considered significant); after being considered significant, they were followed by the pairwise Wilcoxon Mann-Whitney tests using Bonferroni post hoc correction (where *P* < 0.0083 is considered significant for six tests). For the myrosinase activity and gluconsinolates, only four observations were available in each group, and using a Wilcoxon Mann-Whitney test with two groups of four observations each (without ties), the smallest *P*-value that can be obtained is 0.02857, and thus in our data no pairwise comparisons could be found to be significant after Bonferroni correction (which requires *P* < 0.0083 when six comparisons are made). For the glucosinolate–myrosinase products, six observations were available in each group and the multicomView R-package (version 0.1–5) was used to categorize the four groups based on significance, as presented in Fig. 6 (http://cran.r-project.org/ and choose package multcompView).

## Results

### *M. brassicae* larvae show stunted growth on *MINELESS* plants

**Fig. 1.**
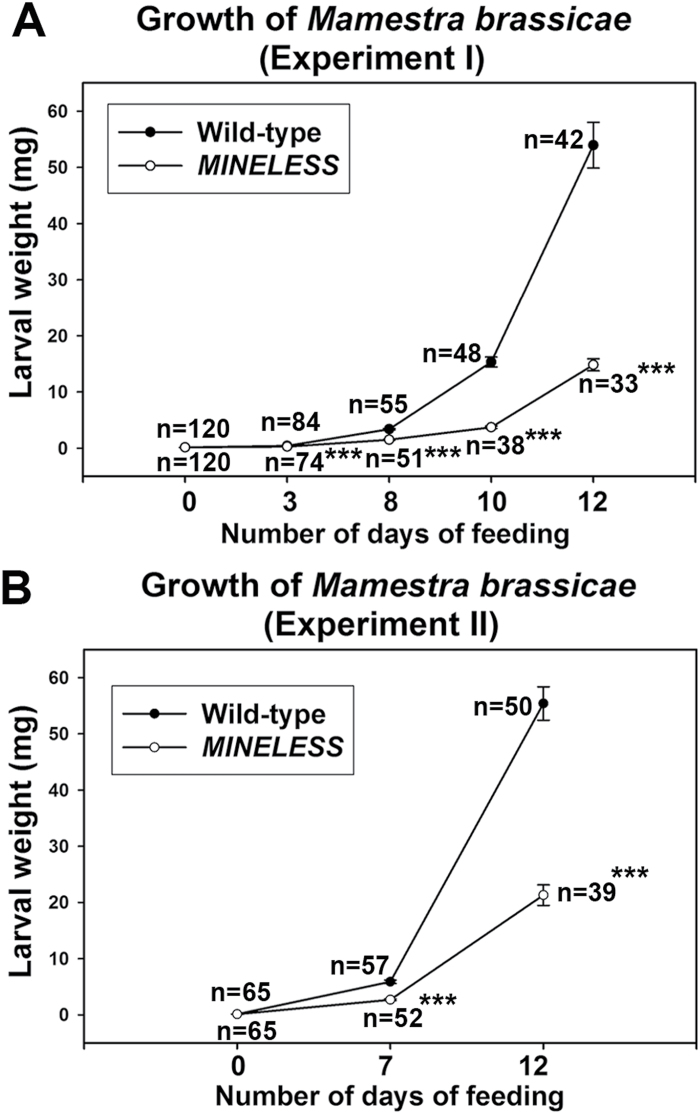
Growth of *M. brassicae* on wild-type and *MINELESS* plants in no-choice feeding experiments. (A, B) The weights of larvae feeding on wild-type and *MINELESS* seedlings differed significantly for day 3, 8, 10, and 12 (A); and day 7 and 12 (B). *n*, number of larvae. Values represent mean ± SE; ***, *P* < 0.001 (Wilcoxon Mann-Whitney test).

The feeding experiments with *M*. *brassicae* showed that larvae gained significantly less weight on *MINELESS* seedlings compared to the wild-type seedlings at all time points: (Experiment I) day 3, 8, 10, and 12, *P* < 0.001; (Experiment II) day 7 and 12, *P* < 0.001 (Fig. 1). The average weights of larvae feeding on *MINELESS* plants were 1.5, 2.2, 4.1, and 3.6 times lower than the average weights of larvae that had been feeding on the wild-type for day 3, 8, 10, and 12, respectively (Experiment I) (Fig. 1A). Similarly, in feeding Experiment II, the average larvae weights were observed to be 2.2 and 2.6 times lower when feeding *MINELESS* seedlings compared to the wild-type for day 7 and 12, respectively (Fig. 1B). Both experiments showed similar reduction (2.2 times) in larvae weights for day 7 (Experiment II) and day 8 (Experiment I). How larvae feed on wild-type and *MINELESS* plants, and how they appeared after 12 days of feeding, can be seen in Supplementary Videos V1 and V2.

**Fig. 2.**
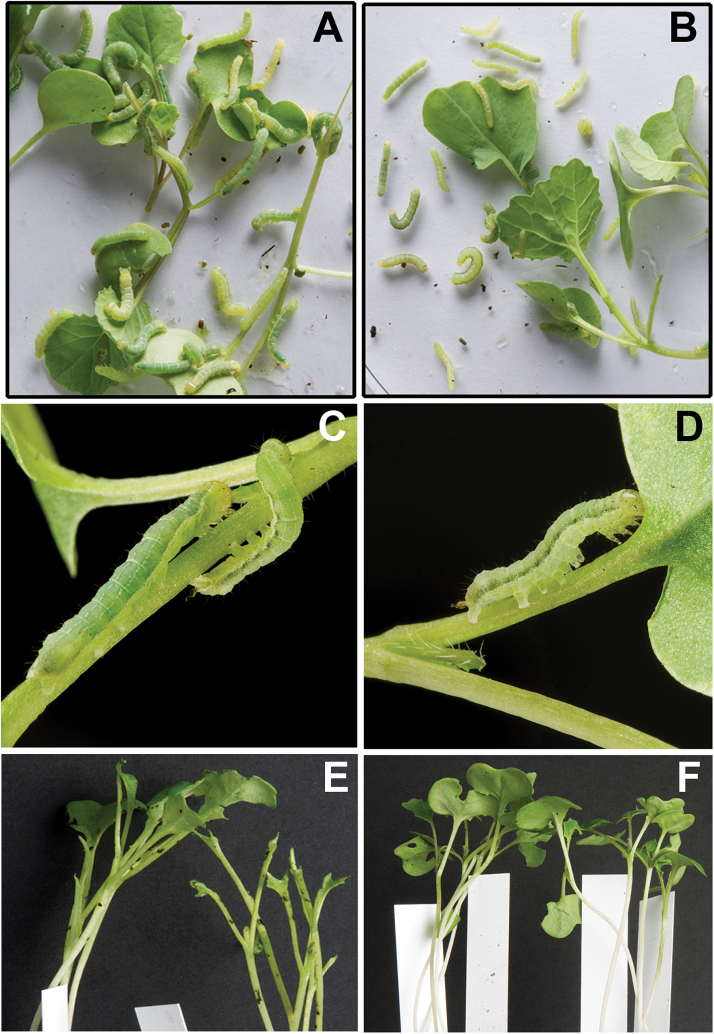
Twelve-day-old *M. brassicae* larvae and *M. brassicae*-damaged wild-type and *MINELESS* seedlings. (A, C) Twelve-day-old larvae that have been feeding from day 0–12 on wild-type plants. (B, D) Twelve-day-old larvae that have been feeding from day 0–12 on *MINELESS* plants. (E, F) *M. brassicae*-damaged wild-type and *MINELESS* plants, respectively.

**Fig. 3.**
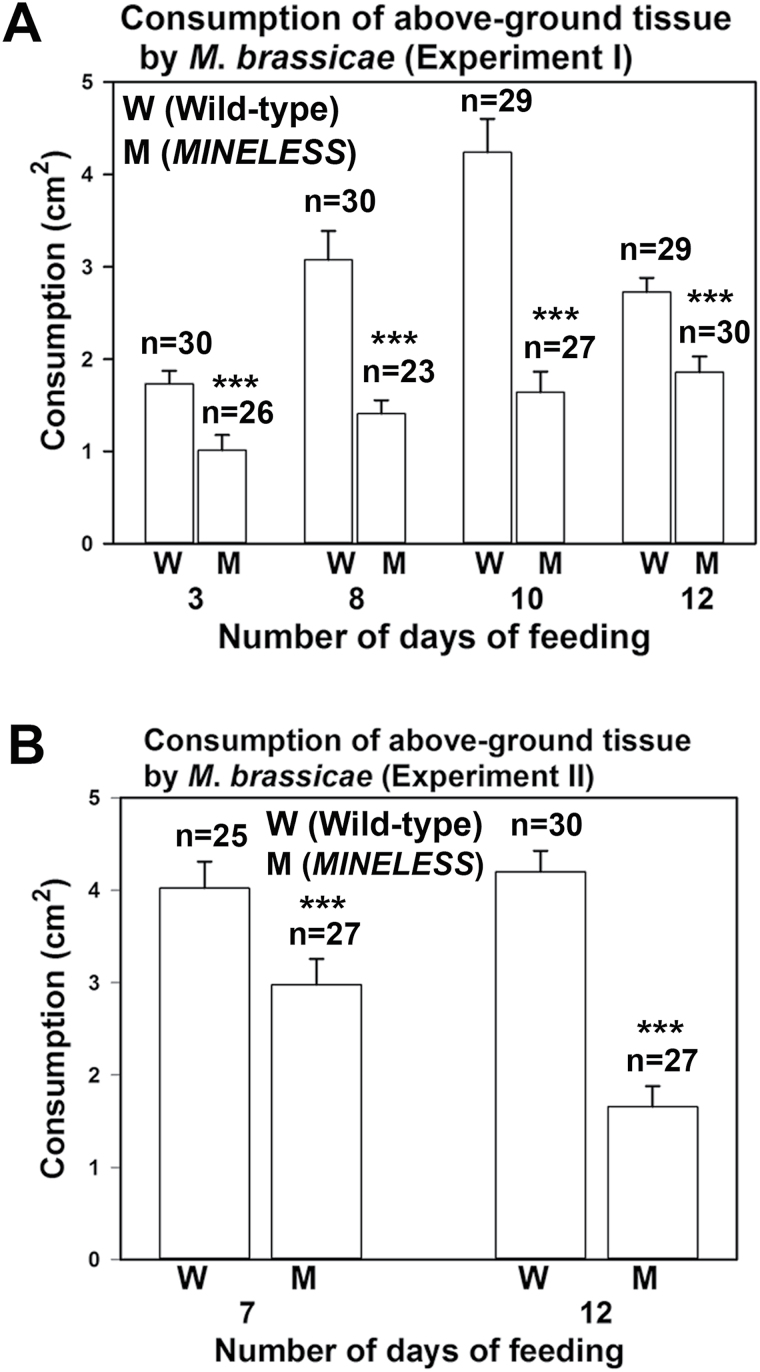
Consumption of wild-type and *MINELESS* seedlings during *M*. *brassicae* no-choice feeding experiments. (A, B) Tissue consumed. *n*, number of seedlings. Values represent mean ± SE; ***, *P* < 0.001; *, *P* < 0.01 (Wilcoxon Mann-Whitney test).

Due to lower weight gain and reduced development, most larvae that had been feeding on *MINELESS* plants appeared smaller than the larvae from wild-type plants (Fig. 2A–D) (Supplementary Videos V1 and V2). Larvae that had been feeding on *MINELESS* plants also showed a greater reduction in number compared to the wild-type (Fig. 1). The larvae also attacked *MINELESS* seedlings less than wild-type seedlings as the *MINELESS* seedlings were consumed less in comparison (Fig. 2E, F) (Fig. 3). This is consistent with the lower weight of larvae feeding on *MINELESS* plants (Fig. 1). The first experiment showed that larvae consumed 41.6%, 54.3%, 61.2%, and 31.9% less *MINELESS* tissue than the wild-type for day 3, 8, 10, and 12, respectively (day 3, 8, 10 and 12; *P* < 0.001) (Fig. 3A) (Supplementary Fig. S1). Similarly, the second experiment showed this consumption to be 26 % and 60.6% less for *MINELESS* compared to the wild-type for day 7 (*P* < 0.0077) and day 12 (*P* < 0.001) (Fig. 3B).

### *MINELESS* plants show no difference for myrosinase activity after *M. brassicae* feeding

**Fig. 4.**
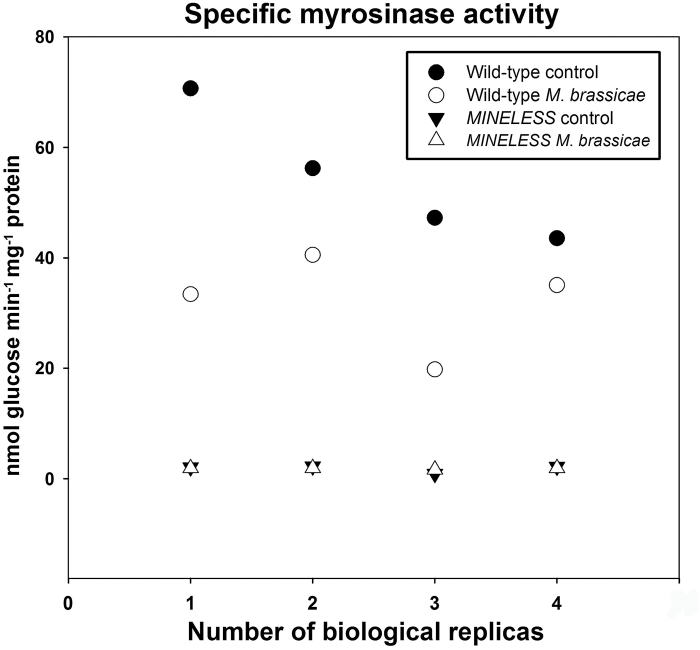
Myrosinase activity in control and *M*. *brassicae*-challenged seedlings (*n* = 4). No pairwise significant differences were found after post hoc Bonferroni corrections (*P* < 0.00833), but a *P* value of 0.02857 was observed for all pairwise comparisons except *MINELESS* control vs *MINELESS M*. *brassicae*.

The Kruskal-Wallis test showed significant differences among the four groups wild-type control, wild-type *M*. *brassicae*, *MINELESS* control, and *MINELESS M*. *brassicae* (*P* < 0.05) (Fig. 4). The myrosinase activity was nearly comparable in *MINELESS* control seedlings and *MINELESS M*. *brassicae* seedlings. The myrosinase activity was very low in *MINELESS* control seedlings (1.85 nmol glucose min^–1^ mg^–1^ protein) compared to the wild-type control seedlings (54.4 nmol glucose min^–1^ mg^–1^ protein). The feeding by larvae of *M*. *brassicae* caused a 41% reduction in myrosinase activity in wild-type seedlings. The pair-wise comparisons of wild-type control vs *MINELESS* control, wild-type control vs wild-type *M*. *brassicae*, and wild-type *M*. *brassicae* vs *MINELESS M*. *brassicae* showed differences with a *P* value of 0.02857, except *MINELESS* control vs *MINELESS M*. *brassicae* (Fig. 4).

### Glucosinolate concentration changes after *M. brassicae* feeding

**Fig. 5.**
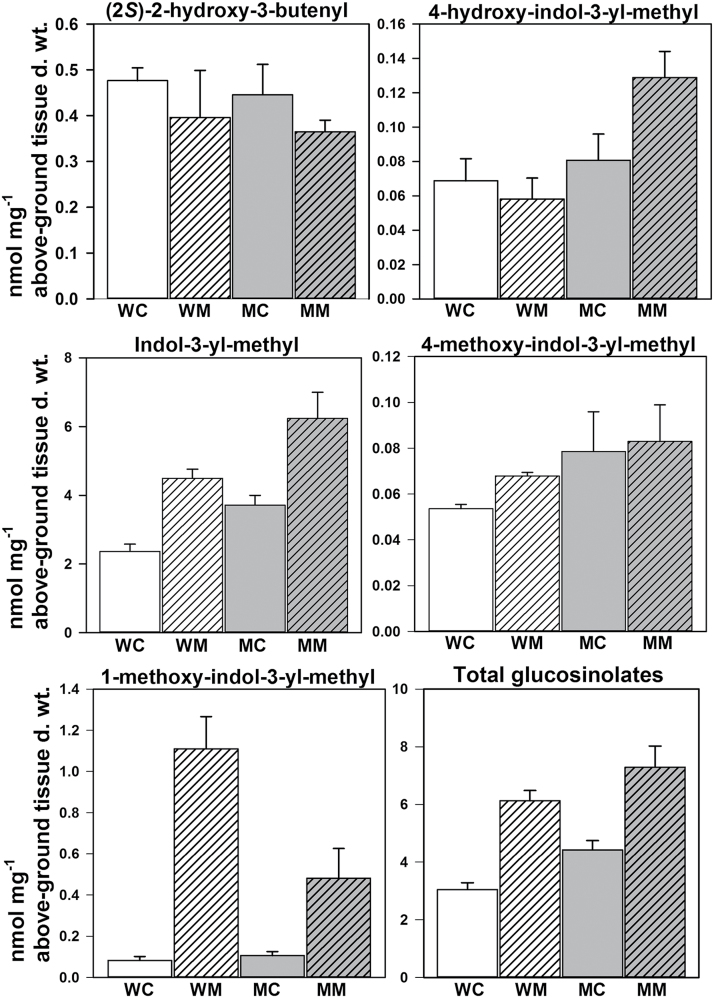
Glucosinolate levels in control and *M*. *brassicae* challenged seedlings (*n* = 4). WC, wild-type control; MC, *MINELESS* control; WM, wild-type *M*. *brassicae*; MM, *MINELESS M*. *brassicae*. Error bars represent SE. No significant differences were found after post hoc Bonferroni corrections (*P* < 0.00833).

Five different glucosinolates were detected in wild-type control as well as *MINELESS* control seedlings (Fig. 5). These five glucosinolates were (2*S*)-2-hydroxy-3-butenyl (epiprogoitrin); and the indole glucosinolates indol-3-yl-methyl (I3M) (glucobrassicin), 4-hydroxy-indol-3-yl-methyl (4OH-I3M) (4-hydroxyglucobrassicin), 4-methoxy-indol-3-yl-methyl (4MO-I3M) (4-methoxyglucobrassicin), and 1-methoxy-indol-3-yl-methyl (1MO-I3M) (neoglucobrassicin). The Kruskal-Wallis test (*P* < 0.05) showed significant differences among the four groups wild-type control, wild-type *M*. *brassicae*, *MINELESS* control, and *MINELESS M*. *brassicae* for 4-hydroxy-I3M, I3M, 1-methoxy-I3M, and total glucosinolates, with levels higher in *MINELESS* control seedlings compared to wild-type control seedlings (Fig. 5).

*M. brassicae* feeding increased I3M, 1MO-I3M, and total glucosinolate levels in both wild-type and *MINELESS* seedlings (*P* = 0.02857); however, 4OH-I3M was observed to be enhanced only in *MINELESS* seedlings (*P* = 0.02857) (Fig. 5). The glucosinolate 4OH-I3M was higher in *MINELESS M*. *brassicae*-challenged seedlings than wild-type *M*. *brassicae*-challenged seedlings (*P* = 0.02857). In contrast, 1MO-I3M was nearly significantly different (*P* = 0.057) in wild-type *M. brassicae-*challenged seedlings and *MINELESS M. brassicae* challenged seedlings.

### Glucosinolate–myrosinase hydrolysis products vary between wild-type and *MINELESS* with and without *M. brassicae* feeding

**Fig. 6.**
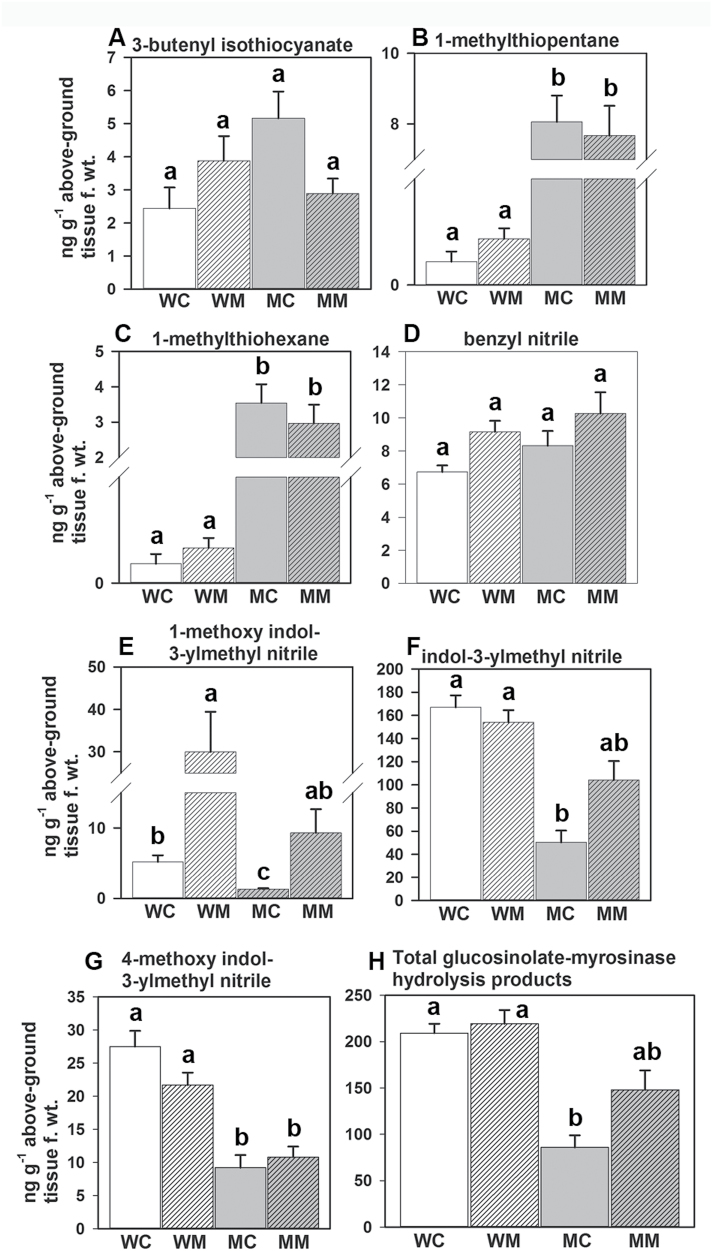
Glucosinolate–myrosinase hydrolysis product levels in control and *M*. *brassicae*-challenged seedlings (*n* = 6). Different letters above the bars indicate significant differences for 3-butenyl isothiocyanate (3BITC), 1-methyl thiopentane (1MTPe), 1-methyl thiohexane (1MTHx), benzyl nitrile (BNIT), 1-methoxy indole-3yl-methylnitrile (1MI3M NIT), indole-3yl-methylnitrile (I3M NIT), 4-methoxy indole-3yl-methylnitrile (4MI3M NIT), and total glucosinolate hydrolysis products levels (Kruskal-Wallis test followed by pairwise Wilcoxon Mann-Whitney tests and Bonferroni post hoc testing, *P* < 0.00833). Error bars represent the SE. WC, wild-type control; MC, *MINELESS* control; WM, wild-type *M*. *brassicae*; MM, *MINELESS M*. *brassicae*.

In total, seven glucosinolate-degradation products could be detected by the solvent extraction of wild-type and *MINELESS* seedlings (Fig. 6). These compounds were 3-butenyl isothiocyanate (3BITC), 1-methyl thiopentane (1MTPe), 1-methyl thiohexane (1MTHx), benzyl nitrile (BNIT), 1-methoxy indole-3-yl-methylnitrile (1MI3M NIT), indole-3-yl-methylnitrile (I3M NIT), and 4-methoxy indole-3-yl-methylnitrile (4MI3M NIT). The Kruskal-Wallis test (*P* < 0.05) showed significant differences among the four groups wild-type control, wild-type *M*. *brassicae*, *MINELESS* control, and *MINELESS M*. *brassicae* for all compounds except 3BITC. The wild-type control and *MINELESS* control seedlings differed significantly for 1MTPe, 1MTHx, I3M NIT, 1MI3M NIT, 4MI3M NIT, and for the total glucosinolate–myrosinase hydrolysis products (*P* < 0.05). I3M NIT, 4MI3M NIT, and total glucosinolate–myrosinase hydrolysis products were low in *MINELESS* control seedlings compared to their wild-type control, while 1MTPe and 1MTHx showed higher levels in *MINELESS* control seedlings (Fig. 6).

*M*. *brassicae* feeding significantly increased levels of 1MI3M NIT (*P* < 0.002) in both wild-type and *MINELESS* seedlings (Fig. 6E). The wild-type *M*. *brassicae*-challenged seedlings differed from *MINELESS M*. *brassicae*-challenged seedlings for 1MTPe, 1MTHx (*P* < 0.002), and 4MI3M NIT (*P* < 0.004) (Fig. 6).

### Gene regulation in wild-type and *MINELESS* plants upon *M. brassicae* attack

Oligonucleotide microarrays from *Arabidopsis* were used and the microarray data were validated by qRT-PCR using *Brassica*-specific sequences corresponding to *Arabidopsis* genes. *B. napus* and *Arabidopsis* are both members of the Brassicaceae family and shared a common ancestor about 13–17 million years ago. This means that orthologous/paralogous gene pairs from these plants have retained high homology to each other even at the DNA level. Previous analyses have shown that 87% of sequences are conserved between *B. napus* and *Arabidopsis* (Cavell *et al.*, 1998). The close evolutionary relationship between *Arabidopsis* and other *Brassica* spp. means that *Arabidopsis* microarrays can be used for gene expression studies of *Brassica* spp. *Arabidopsis* microarrays have been successfully applied and compared for gene expression studies in *Brassica* spp. Examples include studies of insect feeding by *Pieris rapae* in *B*. *oleracea* (Broekgaarden *et al.*, 2007), infestation by *Brevicoryne brassicae* (Broekgaarden *et al.*, 2008), and the identification of plant defence genes in canola (*B*. *napus*) (Schenk *et al.*, 2008).

**Fig. 7.**
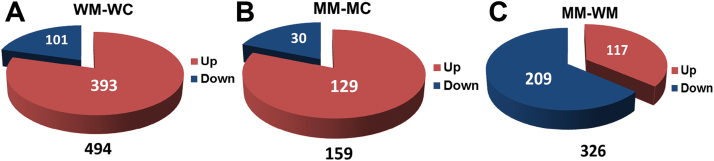
Gene regulation in wild-type and *MINELESS* seedlings after *M*. *brassicae* feeding. (A) Up- and downregulated genes in wild-type *M*. *brassicae* (WM) seedlings vs wild-type control (WC). (B) Up- and downregulated genes in *MINELESS M*. *brassicae* (MM) vs *MINELESS* control (MC). (C) Up- and downregulated genes in *MINELESS* MM after comparison of MM seedlings against WM seedlings.

Transcriptional analysis showed 494 genes to be differentially regulated in wild-type seedlings after *M*. *brassicae* feeding. Out of these 494 genes, 393 were upregulated (log_2_ ratio > 0.5; adjusted *P* ≤ 0.05), and 101 downregulated (log_2_ ratio < –0.5; adjusted *P* ≤ 0.05) (Fig. 7A). In contrast, in *MINELESS* seedlings, after *M*. *brassicae* feeding, only 159 genes were found to be significantly regulated. Out of these 159 genes, 129 showed induction (log_2_ ratio > 0.5; adjusted *P* ≤ 0.05), while 30 genes showed suppression (log_2_ ratio < –0.5; adjusted *P* ≤ 0.05) (Fig. 7B). Comparison of gene expression data of wild-type *M*. *brassicae*-challenged seedlings to *MINELESS M*. *brassicae*-challenged seedlings showed 326 genes to be differentially regulated. Of these, 209 genes showed suppression (log_2_ ratio < –0.4; adjusted *P* ≤ 0.05), while the remaining 117 genes showed induction in *MINELESS* seedlings (log_2_ ratio > 0.4; adjusted *P* ≤ 0.05) (Fig. 7C).

### Expression patterns of genes representing jasmonic acid biosynthesis and signalling pathway

**Fig. 8.**
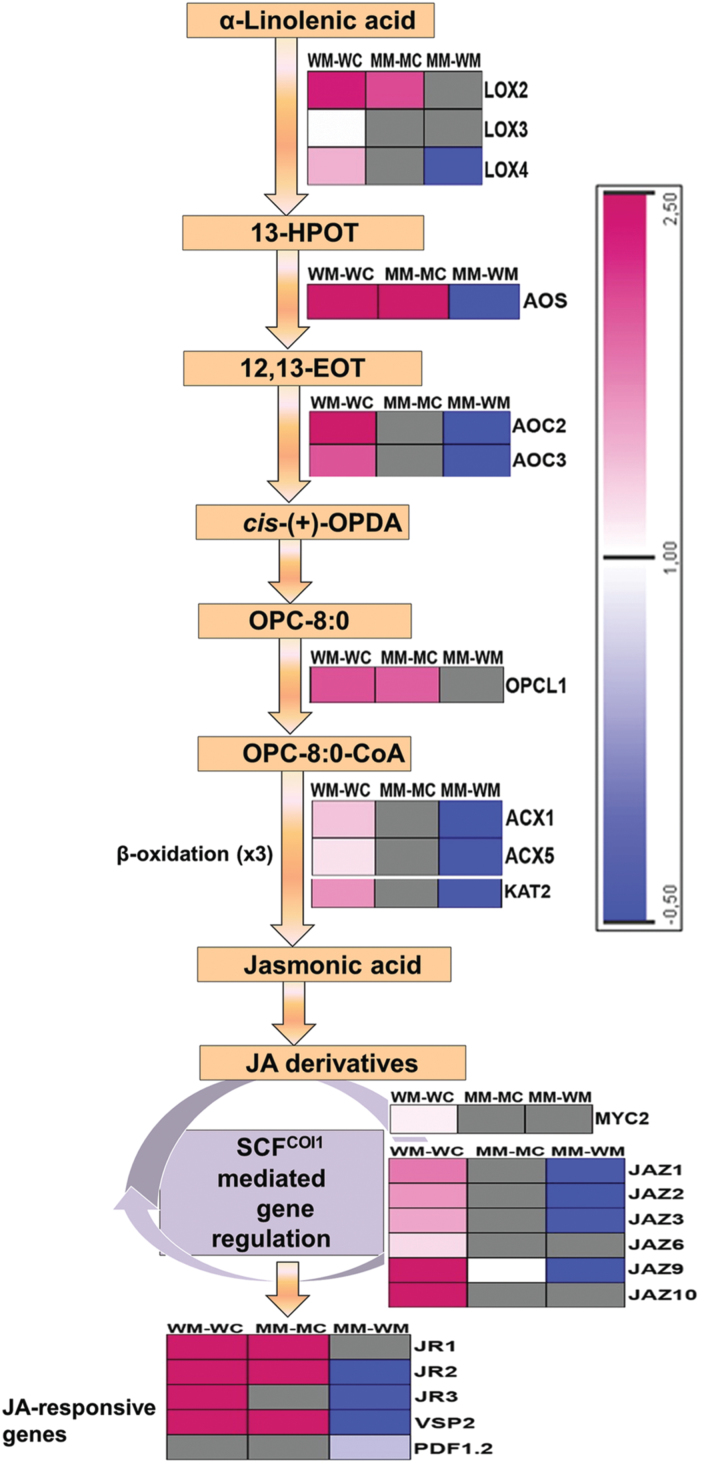
Regulation of genes involved in the jasmonic acid (JA) pathway and JA responsiveness after *M*. *brassicae* feeding. The colour scale represents log_2_-transformed gene expression ratios. The grey boxes represent non-regulated genes. More detailed information about genes is given in Supplementary Tables S4, S5, and S6. Abbreviations: MM-MC, *MINELESS M*. *brassicae* challenged vs *MINELESS* control; WM-WC, wild-type *M*. *brassicae* challenged vs wild-type control; MM-WM, *MINELESS M*. *brassicae* challenged vs wild-type *M*. *brassicae* challenged.

Several genes involved in the jasmonic acid (JA) biosynthesis and signalling pathway were upregulated in wild-type seedlings after *M. brassicae* attack (Fig. 8) (Supplementary Table S4). *B. napus* genes encoding homologues of *Arabidopsis LOX2*, *LOX3*, *LOX4*, *AOS*, *AOC2,* and *AOC3*, all involved in the initiation of JA synthesis in plastids, were upregulated. The gene *OPCL1*, encoding enzyme OPC-8:CoA ligase 1, that esterfies a CoA to the acyl group of OPC-8:0 (Acosta and Farmer, 2010), also showed upregulation in wild-type. Two acyl-coenzyme A oxidase genes, *ACX1* and *ACX5*, and *KAT2*, involved in (the three cycles of) *β*-oxidation leading to the production of JA, were also induced. JA insensitive 1 (*JIN1*/*MYC2*), which regulates the transcription of JA-responsive genes, was also upregulated. Three JA-responsive genes (*JR1*, *JR2*, and *JR3*) and *VSP2* were strongly upregulated. Some of the genes involved in the JA biosynthesis and signalling pathways (*LOX2*, *AOS*, and *OPCL1*) were also upregulated in *MINELESS* seedlings after *M. brassicae* attack, although their regulation was relatively low compared to gene regulation in the wild-type (Fig. 8) (Supplementary Table S5). After *M*. *brassicae* attack on *MINELESS* seedlings, the genes that were most induced were homologues of *AOS, LOX2*, *OPCL1*, *VSP2 JR1*, and *JR2*. Only one gene, encoding *JAZ9*, was detected as induced in *MINELESS* seedlings after *M*. *brassicae* attack compared to the wild-type seedlings, where six *JAZ* genes had showed induction (Fig. 8).

A comparison of *M. brassicae*-challenged *MINELESS* seedlings to *M. brassicae*-challenged wild-type seedlings showed that the JA responses were stronger in wild-type seedlings compared to *MINELESS* seedlings. *B. napus* genes homologous to *LOX4*, *AOS*, *AOC2*, *AOC3*, *OPCL1*, *ACX1*, *ACX5*, *KAT2*, *JAZ1*, *JAZ2*, *JAZ3*, *JAZ9*, *JR2*, *JR3*, and *VSP2* showed lower induction or downregulation in *MINELESS* seedlings compared to the wild-type seedlings. (Fig. 8) (Table S6).

### Expression patterns of genes representing pathways of tryptophan biosynthesis and indole glucosinolate biosynthesis

**Fig. 9.**
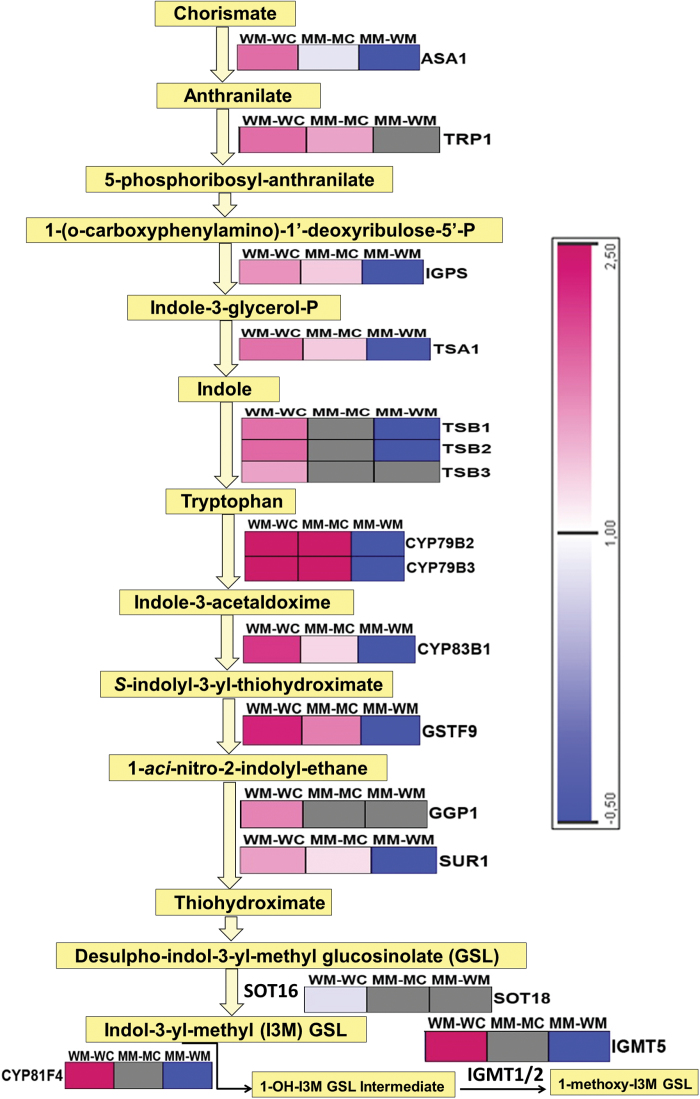
Regulation of genes involved in tryptophan and glucosinolate (GSL) biosynthesis pathways after *M*. *brassicae* feeding. The colour scale represents log_2_-transformed gene expression ratios. The grey boxes represent non-regulated genes. More detailed information about genes is given in Supplementary Tables S4, S5, and S6. Abbreviations: MM-MC, *MINELESS M*. *brassicae* challenged vs *MINELESS* control; WM-WC, wild-type *M*. *brassicae* challenged vs wild-type control; MM-WM, *MINELESS M*. *brassicae* challenged vs wild-type *M*. *brassicae* challenged.

The attack by *M. brassicae* on wild-type plants resulted in upregulation of *B*. *napus* gene homologues of *Arabidopsis ASA1*, *TRP1*, *IGPS*, *TSA1*, *TSB1*, *TSB2*, and *TSB3*, which are involved in tryptophan biosynthesis from chorismate (Fig. 9) (Supplementary Table S4). In contrast, *MINELESS* seedlings only showed induction of *ASA1*, *TRP1*, *IGPS*, and *TSA1* after *M*. *brassicae* attack (Fig. 9) (Supplementary Table S5). The comparison of wild-type *M*. *brassicae*-challenged seedlings to *MINELESS M*. *brassicae-*challenged seedlings showed that genes corresponding to *ASA1*, *IGPS*, *TSA1*, *TSB1*, and *TSB2* were downregulated or less affected in *MINELESS* seedlings (Fig. 9) (Supplementary Table S6).

The following indole glucosinolate core pathway genes showed upregulation in wild-type seedlings upon *M*. *brassicae* feeding: *CYP79B2*, *CYP79B3*, and *CYP83B1*, encoding cytochrome P450 proteins, and *GSTF9*, *GGP1*, and *SUR1*, involved in the biosynthesis of I3M glucosinolate from tryptophan (Fig. 9) (Supplementary Table S6). *CYP81F4* was strongly upregulated in wild-type seedlings after *M*. *brassicae* attack. *CYP81F4* has been reported to be responsible for the production of 1MOI3M glucosinolate (Pfalz *et al.*, 2011), which is produced through a hydroxylation reaction of the glucosinolate indole ring leading from I3M to the 1-hydroxy-indol-3-yl-methyl (1-OH-I3M) glucosinolate intermediate. The 1-OH-I3M glucosinolate intermediate is converted to 1MOI3M by either indole glucosinolate methyltransferase 1 (*IGMT1*) or *IGMT2*. In wild-type seedlings, *IGMT5* was induced after *M*. *brassicae* feeding. *IGMT5* shares 70% of its sequence identity with *IGMT1* and *IGMT2* (Pfalz *et al.*, 2011). The *B*. *napus* homologues of *CYP79B2*, *CYP79B3*, *CYP83B1*, *GSTF9*, and *SUR1*, all key genes in glucosinolate biosynthesis, showed moderate to strong upregulation in *MINELESS* seedlings upon *M*. *brassicae* feeding (Fig. 9) (Supplementary Table S5). The comparison of wild-type *M*. *brassicae*-challenged seedlings to *MINELESS M*. *brassicae-*challenged seedlings showed that *B*. *napus* homologues of *CYP79B2*, *CYP79B3*, *CYP83B1*, *GSTF9*, *SUR1*, *CYP81F4*, and *IGMT5* were downregulated in *MINELESS* seedlings compared to wild-type seedlings (Fig. 9) (Supplementary Table S6).

### Validation of genes belonging to JA biosynthesis and indole glucosinolate pathways using qPCR

**Fig. 10.**
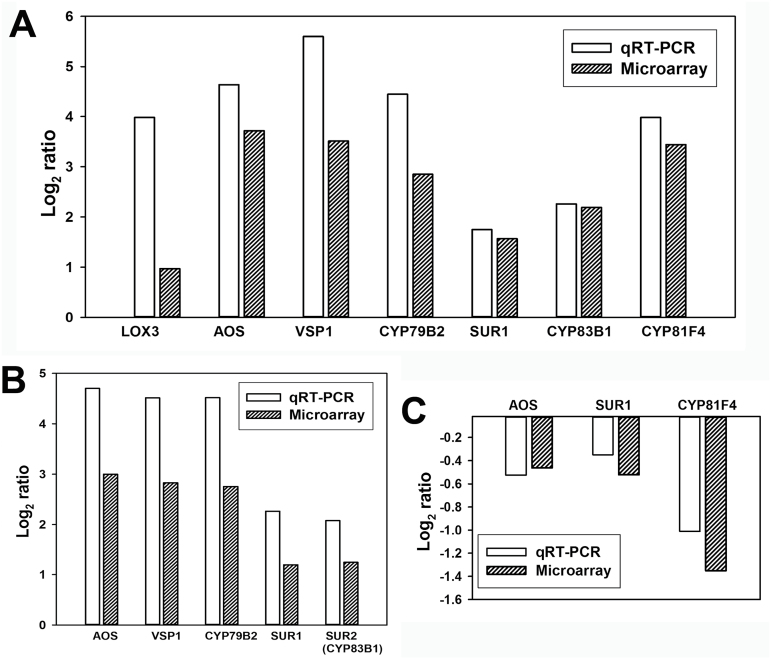
qRT-PCR analyses of genes (log_2_ ratios) belonging to jasmonic acid (JA), and glucosinolate biosynthesis pathways from control and *M*. *brassicae*-challenged seedlings. (A) Wild-type *M. brassicae* vs. wild-type control. (B) *MINELESS M. brassicae vs. MINELESS* control. (C) *MINELESS M.* *brassicae* vs. wild-type M. *brassicae*. Information about *Brassica* genes (with *Arabidopsis* homologues) is given in Supplementary Table S2 and the primer sequences are given in Supplementary Table S3. Transcript levels were normalized to NADH measured in the samples. Values are means of four independent biological replicas.

A subset of genes belonging to JA and glucosinolate pathways that were differentially regulated in wild-type and *MINELESS* seedlings after *M*. *brassicae* feeding were selected for qPCR analysis. Based on the *Arabidopsis* gene information, locus gene IDs, gene sequences, and microarray probe sequences, the corresponding *B. napus* gene homologues were identified, and these DNA sequences were used to design *B. napus*-specific primers (Supplementary Tables S2 and S3). For wild-type *M*. *brassicae*-challenged seedlings, the expression patterns of seven genes, *LOX3*, *AOS*, and *VSP1* (JA-biosynthesis and signalling), *CYP79B2*, *SUR1*, *CYP83B1*, and *CYP81F4* (indole glucosinolate biosynthesis), corresponded well to the profiles obtained from microarray data (Fig. 10A). Similarly, for *MINELESS M*. *brassicae*-challenged seedlings, the expression patterns of five genes, *AOS*, *VSP1*, *CYP79B2*, *SUR1*, and *CYP83B1* matched well with the patterns obtained from microarray data (Fig. 10B). The qRT-PCR validation of three genes from wild-type *M*. *brassicae*-challenged seedlings compared to *MINELESS M*. *brassicae*-challenged seedlings also showed correspondence with gene expression profiles obtained from microarray data (Fig. 10C).

## Discussion

Based on insect no-choice feeding experiments, we found that *M*. *brassicae* (a generalist) larvae show reduced growth and less preference for *MINELESS* seedlings compared to the wild-type seedlings (Fig. 1) (Supplementary videos V1 and V2). Accordingly, we observed that *M*. *brassicae* larvae consumed more of wild-type cotyledons than *MINELESS* seedlings (Figs 2–3, Supplementary Figure S1). Additionally, the data showed a reduction in survival rate of larvae on *MINELESS* compared to the wild-type during their development period from 3 to 12 days (Fig. 1). The *MINELESS* plants seemed to be more resistant to *M*. *brassicae* herbivory compared to the wild-type as larvae either developed more slowly or died on *MINELESS* plants. This could be due to high glucosinolate levels (Fig. 5), or to the presence of new glucosinolate–myrosinase hydrolysis products, 1MTPe and 1MTHx (Fig. 6), which were 36.5- and 38.8-fold higher in the *MINELESS* control compared to the wild-type control. The detection of high amounts of these compounds particularly in *MINELESS* seedlings is interesting as we have not found these compounds before in *MINELESS* seeds or seedlings (Borgen *et al.*, 2010; Ahuja *et al.*, 2011; Borgen *et al.*, 2012). 1 MTPe has been detected in *B*. *napus* and *B*. *oleracea* (Romanesco cauliflower) (Tollsten and Bergström, 1988; Valette *et al.*, 2003), and 1MTHx in seeds of *Prunus domestica* (Ahmed *et al.*, 2007).

Plants show a diverse range of defences that may vary in effectiveness against generalist and specialist insect herbivores (Ahuja *et al.*, 2010; Ali and Agrawal, 2012; Poelman *et al.*, 2008). The results from previous insect feeding experiments with *Arabidopsis* myrosinase mutants seem to vary for generalist and specialist herbivores. In that study, the weight of *Trichoplusia ni* larvae was significantly increased on *tgg1 tgg2* mutants, while the specialist *Pieris rapae* performed better on the wild-type than on *tgg1 tgg2* mutants, which has been considered to be due to reduced feeding stimulants (glucosinolate–myrosinase hydrolysis products) in *tgg1 tgg2* mutants. Our results of challenging myrosinase-mutated *MINELESS* plants to *M*. *brassicae* can be seen as somewhat similar to the response of *P*. *rapae* to *tgg1 tgg2* mutants (Barth and Jander, 2006), as myrosinase-mutated *MINELESS* plants also have reduced feeding stimulants (glucosinolate–myrosinase hydrolysis products) (Ahuja *et al.*, 2011). The authors speculated about the possibility that the *Arabidopsis* double mutant *tgg1 tgg2* could produce another deterrent to compensate for the loss of glucosinolate–myrosinase hydrolysis products (Barth and Jander, 2006). Additionally, it is worth mentioning that even if *M*. *brassicae* is not a specialist, it has some association or preference for cruciferous crops (Bretherton *et al.*, 1979; Rojas *et al.*, 2000; Beekwilder *et al.*, 2008; Ploomi *et al.*, 2009), further highlighting that *M*. *brassicae* may be a specialist for *Brassica* plants and not a generalist. Furthermore, an approximate 4-fold difference in myrosinase activity among lines of *B*. *juncea* decreased feeding by *Plutella xylostella* (a crucifer specialist) on the lines with highest activity relative to the lowest, but there was no difference in feeding by *Spodoptera eridania* (a generalist) (Li *et al.*, 2000). In another similar observation, in *B*. *rapa* populations that were artificially selected for divergent myrosinase levels (~2.5-fold higher), the high myrosinase population was more resistant to the flea beetle *Phyllotreta cruciferae* than the low myrosinase population (Siemens and Mitchell-Olds, 1998). On the other hand, variation in myrosinase levels had no effect on the feeding behaviour of *Brevicoryne brassicae* on *Arabidopsis* (Barth and Jander, 2006) or *Athalia rosae* on *Sinapis alba* (Müller and Sieling, 2006; Travers-Martin and Müller, 2007).

The myrosinase activity in wild-type seedlings was almost in the same range as it has been reported previously for *B*. *napus* hypocotyls (Bones, 1990). In recent years, some non-traditional myrosinases (glucosidases) have been reported to degrade indole glucosinolates (Bednarek *et al.*, 2009; Clay *et al.*, 2009; Bednarek *et al.*, 2011). This indicates that there might also be such glucosidases in *B*. *napus*. If present, where these are potentially acting is still unknown. The standard myrosinase assay revealed low myrosinase activity in *MINELESS*, and analysis of glucosinolate hydrolysis products clearly shows low concentrations, which could be taken as a measure that if such non-traditional myrosinases are present, they are less active than the traditional thioglucosidases.

Myrosinase activity was reduced by *M*. *brassicae* feeding in wild-type seedlings, but not in *MINELESS* seedlings. The latter was not surprising as the myrosinase activity in non-challenged *MINELESS* seedlings is extremely low. Previous studies on myrosinase activity in response to herbivores have also reported a net decline in activity, for example after *A*. *rosae* feeding on *B*. *juncea* (Müller and Sieling, 2006) and *P*. *xylostella* feeding on *B*. *napus* (Pontoppidan *et al.*, 2005). These studies showed decreases in soluble activity, which were also observed for the wild-type after *M*. *brassicae* feeding. On the other hand, some studies revealed induction or increases in myrosinase activity after herbivore attack by *B*. *brassicae* on wild-type and *MINELESS* seedlings (Borgen *et al.*, 2012) and, for example, by *P*. *xylostella* on *B*. *rapa* and by *A*. *rosae* on *S*. *alba* (Martin and Müller, 2007; Travers-Martin and Müller, 2007). Moreover, Textor and Gershenzon mentioned in a review that there is no general induction of myrosinase activity after herbivory, but that it instead triggers the appearance of various associated proteins, the functions of which remain to be explained (Textor and Gershenzon, 2009).

In this study, the total glucosinolate levels were observed to be higher in *MINELESS* control seedlings relative to wild-type control seedlings. Moreover, in our previous studies, we have shown the total glucosinolate levels to be higher in *MINELESS* control (non-infested) seeds than wild-type control seeds and seedlings (Borgen *et al.*, 2010; Ahuja *et al.*, 2011; Borgen *et al.*, 2012). Glucosinolates form a constitutive defence (Wittstock and Gershenzon, 2002), but it is also evident that these compounds accumulate in response to wounding or herbivory (Doughty *et al.*, 1995; Bartlet *et al.*, 1999; Brader *et al.*, 2001; Mikkelsen *et al.*, 2003). A variety of glucosinolates are required to limit the growth of various insect herbivores (Gols *et al.*, 2008; Müller *et al.*, 2010). Gols and colleagues found a negative relationship between the total levels of glucosinolates and survival of *M*. *brassicae* larvae, proposing that *M*. *brassicae* responds to high levels of glucosinolates rather than to specific glucosinolates (Gols *et al.*, 2008). The levels of three glucosinolates (4OH-I3M, I3M, and 4MO-I3M), although non-significant, showed more elevation in *MINELESS* control seedlings compared to the wild-type seedlings. At the same time, *MINELESS* showed low myrosinase activity, which meant that more glucosinolates were intact, giving the seedlings higher glucosinolate levels (Borgen *et al.*, 2010; Ahuja *et al.*, 2011; Borgen *et al.*, 2012), which probably also affects the growth of *M*. *brassicae* larvae. The levels of most of the indole glucosinolates were increased in both wild-type and *MINELESS M*. *brassicae*-challenged seedlings. Similarly, indole glucosinolate biosynthesis genes (*CYP79B2, CYP79B3, SUR2, SUR1, and GSTF9*), also showed upregulation in both wild-type and *MINELESS* after *M. brassicae* feeding. The genes *CYP79B2* and *SUR2,* catalysing the conversion of tryptophan to indole-3-acetaldoxime (IAOx), also showed induction in *B*. *oleracea* and *Arabidopsis* after *P*. *rapae* feeding (Reymond *et al.*, 2004; Broekgaarden *et al.*, 2008). Similar responses for these genes have also been observed in *Arabidopsis* ecotypes upon feeding by aphids *Brevicoryne brassicae* and *Myzus persicae* (Kusnierczyk *et al.*, 2007). An induction of genes of the indole glucosinolate pathway, and of indole glucosinolates levels, has frequently been reported as a herbivore response in Brassicaceae and the model plant *Arabidopsis* (Bodnaryk, 1992; Hopkins *et al.*, 1998; Bartlet *et al.*, 1999; Kusnierczyk *et al.*, 2007; Kusnierczyk *et al.*, 2008; Poelman *et al.*, 2008; Textor and Gershenzon, 2009). *CYP81F4* (strongly induced in wild-type seedlings) has been shown to be mainly responsible for the production of 1MO-I3M (Pfalz *et al.*, 2011) (Figs. 9 and 10A). Our results showed a corresponding increase in glucosinolate 1MO-I3M in wild-type seedlings after *M*. *brassicae* feeding (Fig. 5), thereby supporting the role of *CYP81F4* in production of glucosinolate 1MO-I3M. A very similar trend was also observed for its hydrolysis product 1MI3M NIT (Fig. 6E).

The induction of JA synthesis and signalling pathways, and JA responsive genes, is a well known response to insect attack (Acosta Iván and Farmer Edward, 2010; Ballaré, 2011; Verhage *et al.*, 2011). Genes involved in the biosynthesis of JA are upregulated after feeding by insect herbivores such as *P*. *rapae* and *P. xylostella* (Reymond *et al.*, 2004; Broekgaarden *et al.*, 2007; Ehlting *et al.*, 2008; Kusnierczyk *et al.*, 2011), and JA has been shown to be responsible for *Arabidopsis* resistance to cabbage looper (*T. ni*) (Chehab *et al.*, 2011). It is therefore not surprising that key genes involved in JA synthesis and signalling, such as *LOX2*, *AOS*, *AOC2*, *OPCL1*, *OPR1*, *ACX1*, *KAT1, MYC2,* and several JAZs, were upregulated in wild-type seedlings after *M*. *brassicae* feeding. However, in *MINELESS* seedlings after *M*. *brassicae* feeding, only a few genes (*LOX2*, *AOS*, *OPCL1*, and *JAZ9*) showed upregulation with relatively low induction levels compared to the wild-type (Fig. 8). The regulation of fewer genes of the JA pathway, and their low expression levels in *MINELESS* compared to the wild-type, could be due to the reduced feeding by *M*. *brassicae* larvae on *MINELESS* plants. However, we cannot rule out the possibility that it could also be due to lower levels of myrosinase and glucosinolate–myrosinase hydrolysis products, leading to reduced induction of genes in the JA-signalling pathway in *MINELESS* plants after attack by *M*. *brassicae* larvae.

## Conclusions and perspectives

Plant–insect interactions have been studied using the insect herbivore cabbage moth (*M. brassicae*), and wild-type and *MINELESS B. napus* plants that lack plant defence cells called myrosin cells, also known as the toxic mustard oil mines. The results showed that *M*. *brassicae* larvae chewed more and performed better on wild-type *B*. *napus* plants than on *MINELESS B*. *napus* plants. The reduced performance of *M*. *brassicae* larvae on *MINELESS* seedlings is possibly due to the higher levels of indole- and total glucosinolates in *MINELESS* control (non-infested) seedlings. Due to the reduction in myrosinase levels, *MINELESS* plants have reduced amounts of glucosinolate hydrolysis products. However, the glucosinolate–myrosinase hydrolysis products, 1MTPe and 1MTHx, were observed in very high amounts in *MINELESS* compared to the wild-type seedlings, which might be affecting preference or feeding behaviour. The results also highlight that *M*. *brassicae*, which is a generalist herbivore but has some preference for Brassicaceae plants, can be a specialist for brassicas and not a generalist. As expected, the transcriptional responses showed JA as the key mediator of the defence response towards insect herbivory as several genes involved in the JA biosynthesis pathway, signalling, and JA responsiveness were upregulated in both wild-type and *MINELESS* seedlings. The genes belonging to tryptophan biosynthesis and indole glucosinolate pathway genes were induced, and the indole glucosinolate levels were elevated by *M. brassicae* feeding in both types of plants. A much higher induction of 1MO-I3M glucosinolate was observed for *M. brassicae* in wild-type plants compared with *MINELESS* plants. The comparison of wild-type *M*. *brassicae* and *MINELESS M*. *brassicae*-challenged seedlings showed a number of genes for JA biosynthesis, signalling, and JA-responsiveness, and tryptophan and glucosinolate biosynthesis, to be downregulated in *MINELESS* seedlings. The downregulation of genes *ASA1, IGPS, TSA1, TSB1, TSB2, CYP79B2, CYP79B3, CYP83B1, GSTF9, and SUR1* in MINELESS *brassicae*-challenged seedlings compared to the wild-type *M*. *brassicae*-challenged seedlings probably leads to lower accumulation of the glucosinolates indolyl-3-yl-methyl, 1-methoxy-indol-3-yl-methyl, and 4-methoxy-indol-3-yl-methyl with respect to their control (non-infested) seedlings. Moreover, the induction of fewer genes and lower expression levels in *MINELESS* after *M*. *brassicae* feeding compared to the wild-type could be due to less feeding by *M*. *brassicae* larvae in comparison to the wild-type, which needs to be explored further. Currently, we are using *MINELESS* plants as a representative model for studying defence responses against other insect herbivores, including both generalists and specialists, and above- and below-ground herbivores, to get an overview about the role of myrosin cells in plant–insect interactions. We think that performing such studies will provide more information about the importance of plant defence cells in Brassicaceae plants.

## Supplementary material

Supplementary data can be found at *JXB* online.

Supplementary Table S1. Time-line of insect no-choice and induction experiments.

Supplementary Table S2. Selected *B. napus* genes confirmed by qRT-PCR based on differential regulation in microarray results.

Supplementary Table S3. Primer sequences.

Supplementary Table S4. Regulation of JA biosynthesis, signalling, and JA-responsive genes; and tryptophan and glucosinolate biosynthesis pathway genes. This is in wild-type *M. brassicae*-challenged seedlings, after comparison of these to wild-type control seedlings.

Supplementary Table S5. Regulation of JA biosynthesis, signalling, and JA- responsive genes; and tryptophan and glucosinolate biosynthesis pathway genes. This is in *MINELESS M. brassicae-*challenged seedlings, after comparison of these to *MINELESS* control seedlings

Supplementary Table S6. Regulation of JA biosynthesis, signalling, and JA- responsive genes; and tryptophan and glucosinolate biosynthesis pathway genes. This is in *MINELESS M*. *brassicae-*challenged seedlings, after comparison of wild-type *M*. *brassicae*-challenged seedlings to *MINELESS M*. *brassicae*-challenged seedlings

Supplementary Figure S1. Control (non-infested) and *M*. *brassicae*-damaged cotyledons of wild-type and *MINELESS* seedlings from no-choice feeding experiments. (A, E, I, M) Wild-type control cotyledons from day 3, 8, 10, and 12, respectively. (B, F, J, N) Wild-type damaged cotyledons from day 3, 8, 10, and 12, respectively. (C, G, K, O) *MINELESS* control cotyledons from day 3, 8, 10, and 12, respectively. (D, H, L, and P) *MINELESS* damaged cotyledons from day 3, 8, 10, and 12, respectively.

Supplementary Video S1. *M*. *brassicae* larvae feeding on wild-type plants.

Supplementary Video S2. *M*. *brassicae* larvae feeding on *MINELESS* plants.

## Funding

This work was funded by the RCN grants 185173/V40, 175691/I10 and 184146/S10.

## Supplementary Material

Supplementary Data
