# Supplementary material for: Plant defence responses in oilseed rape MINELESS plants after attack by the cabbage moth Mamestra brassicae
Source: J Exp Bot. 2015 Jan 6;66(2):579–92. doi: 10.1093/jxb/eru490 (PMC4286410; doi:10.1093/jxb/eru490)
Supplement: Supplementary Data [file supp_66_2_579__index.html]

Plant defence responses in oilseed rape MINELESS plants after attack by the cabbage moth Mamestra brassicae — Supplementary Data 

# Plant defence responses in oilseed rape *MINELESS* plants after attack by the cabbage moth *Mamestra brassicae*

## Supplementary Data

Data files

**Files in this Data Supplement:**

- Supplementary Data - Supplementary Data
- Supplementary Data - Supplementary Data
- Supplementary Data - Supplementary Data
- Supplementary Data - Supplementary Data
- Supplementary Data - Supplementary Data
