# Supplementary material for: Plant defence responses in oilseed rape MINELESS plants after attack by the cabbage moth Mamestra brassicae
Source: J Exp Bot. 2015 Jan 6;66(2):579–92. doi: 10.1093/jxb/eru490 (PMC4286410; doi:10.1093/jxb/eru490)
Supplement: Supplementary Data [file supp_eru490_jexbot139121_file005.pdf]

Plant defence responses in oilseed rape MINELESS plants after attack by cabbage moth *Mamestra brassicae*. Ishita Ahuja, Nicole van Dam, Per Winge, Marianne Traelnes, Aysel Heydarova, Jens Rohloff, Mette Langaas, and Atle M Bones

SUPPLEMENTARY DATA

Supplementary Table S1 An overview of time-line of insect no-choice and induction experiments

**Day**	**Event**
0	*Mamestra brassicae* neonate larvae were placed on cotyledons of wild-type and *MINELESS* seedlings (Experiments I and II).
3, 8, 10 and 12	*M. brassicae* larvae were removed and weighed (Experiment I).
3, 8, 10 and 12	*M. brassicae* larvae were given new 7-9 days old seedlings (Experiment I).
7 and 12	*M. brassicae* larvae were removed and weighed (Experiment II).
7 and 12	*M. brassicae* larvae were given new 7-9 days old seedlings (Experiment II).
10	For the induction experiment, 10-days old *M. brassicae* larvae were kept on 6-7 old seedlings of wild-type and *MINELESS*.
11	*M. brassicae* larvae were removed and the above-ground tissue from control (non-infested) and *M. brassicae* challenged seedlings was harvested and frozen in liquid nitrogen. For analysis of glucosinolate-myrosinase hydrolysis products, the fresh tissue was used.

Supplementary Table S2 Selected *Brassica napus* genes confirmed by qRT-PCR, based on the differential regulation in microarray results

***Brassica napus* gene**	***Arabidopsis thaliana* homolog**
LOX3	Lipoxygenase 3 (At1g17420)
AOS	Allene oxide synthase (At5g42650)
VSP1 (VSP2)	Vegetative storage protein 2 (At5g24770)
SUR1	Superroot 1(At2g20610)
CYP79B2	Cytochrome P450 79B2 (At4g39950)
CYP83B1(SUR2)	Cytochrome P450 83B1 (At4g31500)
CYP81F4	Cytochrome P450 81F4 (At4g37410)

Supplementary Table S3 Primer sequences

**Gene**	**Sequence, displayed from 5' to 3' end:**
BnNADH	AGATTTGAAGACTCTGAACCCT (Forward)
BnNADH	AATCTGTGACTCGTTCATACCA (Reverse)
BnCYP79B2	ACCCATTGCTTACCGCTGATGA (Forward)
BnCYP79B2	TCCATTGCCTTACGGAGTATCT (Reverse)
BnCYP83B1	TCGAACCAGTCATCCCAATTC (Forward)
BnCYP83B1	TGCGTTCACCTGAATGATGGT (Reverse)
BnCYP81F4	ATCCAGAGTATTACAGTGAGGT (Forward)
BnCYP81F4	ACTCTATTGTCACGGCTGATGT (Reverse)
BnSUR1	GGAAAACCTCGTGTTTCTACCA (Forward)
BnSUR1	TCTCTCAAGTGCATCCTCAAGC (Reverse)
BnVSP1	GATCTTTGACCTAGACGAGACC (Forward)
BnVSP1	TGTAAAGATGCAAAGTCTCTGG (Reverse)
BnAOS	TCAGGAGGTATGATTCCTTTGA (Forward)
BnAOS	CTCAGAGACGTGAATGTAACGG (Reverse)
BnLOX3	TGATGCGACGGTTAATCCCTGA (Forward)
BnLOX3	TTCGACGTTTGCAACAAACTCG (Reverse)

Supplementary Table S4 Regulation of jasmonic acid (JA) biosynthesis, signalling and JA-responsive, tryptophan and glucosinolate biosynthesis pathway genes in wild-type *M. brassicae* (WM) challenged seedlings after comparison of wild-type *M. brassicae* (WM) challenged seedlings to wild-type control (WC) seedlings

Accession	Gene name	Gene description	Log_2	Adj. *P*-value ≤
**1 Jasmonic acid (JA)- biosynthesis, signalling and JA responsive**				
At3g45140	LOX2	Lipoxygenase 2	2.334	0.001
At1g17420	LOX3	Lipoxygenase 3	0.967	0.05
At1g72520	LOX4	Lipoxygenase 4	1.460	0.05
At5g42650	AOS	Allene oxide synthase	3.716	0.001
At3g25770	AOC2	Allene oxide cyclase 2	2.756	0.001
At3g25780	AOC3	Allene oxide cyclase 3	2.013	0.001
At1g20510	OPCL1	OPC-8:0 Co A ligase 1	2.030	0.001
At4g16760	ACX1	Acyl-coenzyme A oxidase 1	1.364	0.01
At2g35690	ACX5	Acyl-coenzyme A oxidase 5	1.177	0.01
At2g33150	KAT2	3-Ketoacyl-Co A thiolase 2	1.650	0.001
At1g32640	MYC2	Jasmonate insensitive 1	1.116	0.05
At1g19180	JAZ1	Jasmonate ZIM-domain 1	1.799	0.001
At1g74950	JAZ2	Jasmonate ZIM-domain 2	1.637	0.01
At3g17860	JAZ3	Jasmonate ZIM-domain 3	1.526	0.01
At1g72450	JAZ6	Jasmonate ZIM-domain 6	1.224	0.01
At1g70700	JAZ9	Jasmonate ZIM-domain 9	2.804	0.001
At5g13220	JAZ10	Jasmonate ZIM-domain 10 (Marker Gene)	3.276	0.001
At3g16470	JR1	Jasmonate responsive 1	3.015	0.001
At4g23600	JR2	Jasmonate responsive 2 / Coronatine induced 1	3.137	0.001
At1g51760	JR3	Jasmonate responsive 3	2.480	0.001
At5g24770	VSP2	Vegetative Storage protein 2 (Marker Gene)	3.511	0.001
**2 Tryptophan and glucosinolate biosynthesis pathway**				
At5g05730	ASA1	Anthranilate synthase component 1	1.861	0.001
At5g17990	TRP1	Tryptophan biosynthesis 1	1.853	0.001
At2g04400	IGPS	Indole-3-glycerol phosphate synthase	1.647	0.001
At3g54640	TSA1	Tryptophan synthase alpha chain 1	1.834	0.001
At5g54810	TSB1	Tryptophan synthase beta chain 1	1.839	0.001
At4g27070	TSB2	Tryptophan synthase beta chain 2	1.896	0.001
At5g28237	TSB3	Tryptophan synthase beta-subunit 3	1.550	0.05
At4g39950	CYP79B2	Cytochrome P450 79B2	2.850	0.001
At2g22330	CYP79B3	Cytochrome P450 79B3	2.990	0.001
At4g31500	CYP83B1/SUR2	Cytochrome P450 83B1	2.188	0.001
At2g30860	GSTF9	Glutathione S-transferase PHI 9	2.305	0.001
At1g69930	GSTU11	Glutathione S-transferase TAU 11	2.300	0.001
At4g30530	GGP1	Gamma-Glutamyl Peptidase 1	1.728	0.001
At2g20610	SUR1	Superroot 1	1.566	0.001
At1g74090	SOT18	Desulphoglucosinolate sulphotransferase 18	0.774	0.05
At4g37410	CYP81F4	Cytochrome P450 81F4	3.442	0.001
At1g76790	IGMT 5	Indole glucosinolate methyltransferase 5	3.590	0.001

Supplementary Table S5 Regulation of jasmonic acid (JA) biosynthesis, signalling and JA-responsive, tryptophan and glucosinolate biosynthesis pathway genes in *MINELESS M. brassicae* challenged seedlings after comparison of *MINELESS M. brassicae* (MM) challenged seedlings to *MINELESS* control (MC) seedlings

**Accession**	**Gene name**	**Gene description**	**Log$_2$**	**Adj. *P*-value ≤**
**1 Jasmonic acid (JA)- biosynthesis, signalling and JA responsive**				
At3g45140	LOX2	Lipoxygenase 2	2.069	0.001
At5g42650	AOS	Allene oxide synthase	2.999	0.001
At1g20510	OPCL1	OPC-8:0 Co A Ligase 1	1.954	0.001
At1g70700	JAZ9	Jasmonate ZIM-domain 9	1.008	0.001
At3g16470	JR1	Jasmonate responsive 1	2.597	0.001
At4g23600	JR2	Jasmonate responsive 2	2.580	0.001
At5g24770	VSP2	Vegetative storage protein 2	2.830	0.001
**2 Tryptophan and glucosinolate biosynthesis pathway**				
At5g05730	ASA1	Anthranilate synthase component 1	0.812	0.05
At5g17990	TRP1	Tryptophan synthase alpha chain 1	1.546	0.001
At2g04400	IGPS	Indole-3-glycerol phosphate synthase	1.316	0.001
At3g54640	TSA1	Tryptophan synthase alpha chain 1	1.317	0.001
At4g39950	CYP79B2	Cytochrome P450 79B2	2.755	0.001
At2g22330	CYP79B3	Cytochrome P450 79B3	2.691	0.001
At4g31500	CYP83B1/SUR2	Cytochrome P450 83B1	1.244	0.001
At2g20610	SUR1	Rooty / Superroot 1; also in adventitious root development	1.195	0.001
At2g30860	GSTF9	Glutathione S-transferase PHI 9	1.762	0.001

Supplementary Table S6 Regulation of jasmonic acid (JA) biosynthesis and signalling, tryptophan and glucosinolate biosynthesis pathway genes in *MINELESS M. brassicae* challenged seedlings after comparison of wild-type *M. brassicae* (WM) challenged seedlings to *MINELESS M. brassicae* (MM) challenged seedlings

**Accession**	**Gene name**	**Gene description**	**Log_2**	**Adj. *P*-value ≤**
**1 Jasmonic acid (JA)- biosynthesis, signalling and JA responsive**				
At5g42650	AOS	Allene oxide synthase	-0.462	0.001
At3g25770	AOC2	Allene oxide cyclase 2	-0.890	0.001
At3g25780	AOC3	Allene oxide cyclase 3	-0.468	0.001
At4g16760	ACX 1	Acyl-coenzyme A oxidase 1	-0.598	0.01
At2g35690	ACX5	Acyl-coenzyme A oxidase 5	-0.456	0.001
At2g33150	KAT2	3-Ketoacyl-CoA thiolase 2	-0.433	0.001
At1g19180	JAZ1	Jasmonate ZIM-domain 1	-0.435	0.001
At1g74950	JAZ2	Jasmonate ZIM-domain 2	-0.653	0.01
At3g17860	JAZ3	Jasmonate ZIM-domain 3 / Jasmonate-insensitive 3	-0.425	0.01
At1g70700	JAZ9	Jasmonate ZIM-domain 9	-0.908	0.001
At4g23600	JR2	Jasmonate responsive 2	-0.570	0.001
At1g51760	JR3	Jasmonate responsive 3	-0.427	0.001
At5g24770	VSP2	Vegetative Storage Protein 2	-0.856	0.001
At5g44420	PDF1.2	Plant defensin 1.2	0.526	0.05
**2 Tryptophan and glucosinolate biosynthesis**				
At5g05730	ASA1	Anthranilate synthase component 1	-0.689	0.001
At2g04400	IGPS	Indole-3-glycerol phosphate synthase	-0.636	0.01
At3g54640	TSA1	Tryptophan synthase alpha chain 1	-0.450	0.001
At5g54810	TSB1	Tryptophan synthase beta chain 1	-0.672	0.001
At4g27070	TSB2	Tryptophan synthase beta chain 2	-0.667	0.001
At4g39950	CYP79B2	Cytochrome P450 79B2	-0.644	0.001
At2g22330	CYP79B3	Cytochrome P450 79B3	-0.694	0.001
At2g20610	CYP83B1/SUR2	Cytochrome P450 83B1	-1.074	0.001
At2g30860	GSTF9	Glutathione S-Transferase PHI 9	-0.490	0.001
At4g31500	SUR1	Superroot 1	-0.522	0.001
At4g37410	CYP81F4	Cytochrome P450 81F4	-1.351	0.001
At1g76790	IGMT5	Indole glucosinolate methyltransferase 5	-0.765	0.001
