# Supplementary figures and images for: Plant defence responses in oilseed rape MINELESS plants after attack by the cabbage moth Mamestra brassicae
Source: J Exp Bot. 2015 Jan 6;66(2):579–92. doi: 10.1093/jxb/eru490 (PMC4286410; doi:10.1093/jxb/eru490)

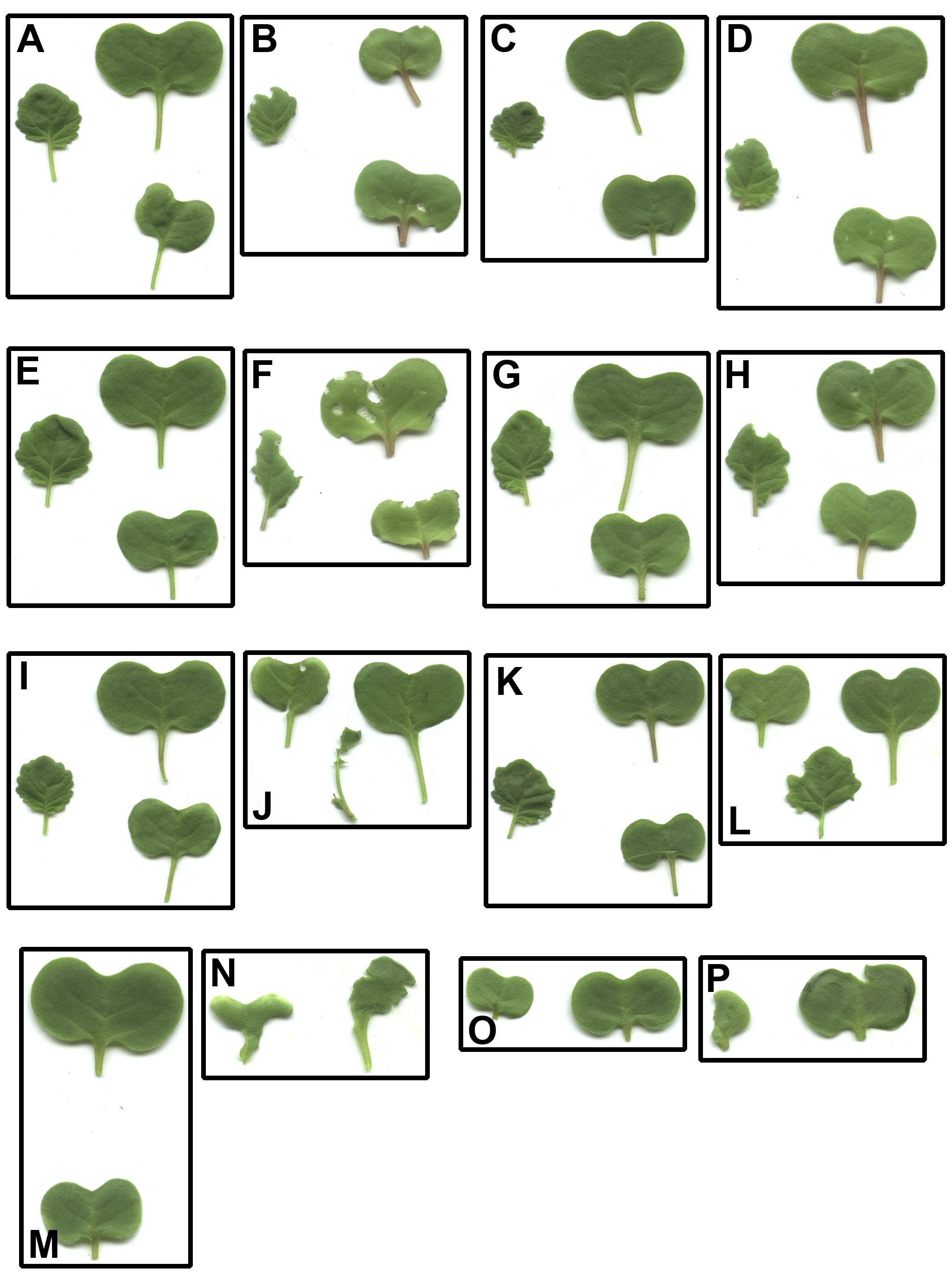

Supplement: Supplementary Data [file supp_eru490_jexbot139121_file001.jpg]
